# Natural history of *SPTBN4*-related neurodevelopmental disorder with hypotonia, neuropathy, and deafness

**DOI:** 10.1186/s13023-025-03810-4

**Published:** 2025-08-08

**Authors:** Hanan AlQudairy, Mohammad A. AlMuhaizea, Mohamed Tohary, Maissa Alfuraih, Aisha Alnafisah, Aljouhra AlHargan, Anoud Albader, Hadeel Jaber, Rawan Almass, Albandary Albakheet, Terfa Alsheddi, Eman AlObeid, Maha M. Alrasheed, Ali Al-Odaib, Hamad AlZaidan, Moeenaldeen D. AlSayed, Namik Kaya

**Affiliations:** 1 NeuroGenetics Unit, Translational Genomics Department, MBC: 26, Genomic Medicine Center of Excellence, King Faisal Specialist Hospital and Research Center, 11211 Riyadh, Saudi Arabia; 2 Neuroimmunology and Neuromuscular Department, MBC:76, Neuroscience Center of Excellence, King Faisal Specialist Hospital and Research Center, Riyadh, Saudi Arabia; 3 College of Medicine, Alfaisal University, P.O. Box 50927, Riyadh, 11533 Saudi Arabia; 4 Department of Medical Genomics, MBC:75, Genomic Medicine Center of Excellence, King Faisal Specialist Hospital and Research Center, P.O. Box 3354, Riyadh, 11211 Saudi Arabia; 5 Department of Clinical Pharmacy, College of Pharmacy, King Saud University, Riyadh, Saudi Arabia; 6 Research Administrative Operations Department, MBC: 03, Research and Innovation, King Faisal Specialist Hospital and Research Center, Riyadh, 11211 Saudi Arabia; 7 King Salman Center for Disability Research, Riyadh, Saudi Arabia

**Keywords:** *SPTBN4*, Autosomal recessive cerebellar ataxia, Splicing variant, Whole exome sequencing, Sanger sequencing, NEDHND

## Abstract

**Background:**

Pathogenic variants in *SPTBN4* have been linked to autosomal recessive “neurodevelopmental disorder with hypotonia, neuropathy, and deafness” (MIM# 617519) known as NEDHND. The disorder is highlighted with neuropathy, muscle weakness, and infrequent appearance of seizures in the affected individuals. This study aims to investigate the natural history of the disease, present genetic and clinical appearance of the syndrome in a highly consanguineous population, Saudi Arabia, and finally provide an overview of the reported cases, their clinical features, and disease-causing variants.

**Methods:**

The study started with a search through neurology clinics and local databases and utilized genetic testing records after diagnosing a patient with NEDHND at our hospital (King Faisal Specialist Hospital and Research Centre, KFSHRC). Based on the search we have identified additional patients (in total, *n* = 10) with the disease and performed genetic testing using whole exome sequencing and confirmatory Sanger sequencing. We performed RT-PCR on RNA extracted from lymphoblastoid cell line from a patient who found to have an aberrant splicing variant. Finally, we comprehensively reviewed current literature and available data related to the disease.

**Results:**

We present natural history of SPTBN4-associated neurodevelopmental disorder with hypotonia, neuropathy, and deafness in addition to four Saudi families with ten affected individuals who share clinical features of NEDHND. We report three known mutations and one novel nonsense variant, highlight atypical clinical features related to cerebellar involvement, confirm the pathogenicity of a splicing variant by RT-PCR, and review the findings of previously reported patients.

**Conclusion:**

Our study defines the clinical phenotype of a cohort of NEDHND in detail including the evolution of patients’ clinical features, compares them to previously reported cases, and utilizes the existing data on the disease to direct development of a better prevention plan by means of genetic and preimplantation counseling. Our study may help and enable future clinical trials focusing on NEDHND in our country.

**Supplementary Information:**

The online version contains supplementary material available at 10.1186/s13023-025-03810-4.

## Introduction

Autosomal recessive genetic disorders with neurodevelopmental manifestations are predicted to be common in consanguineous populations [1]. Many of these genetic disorders present early in life either leading to a premature death or a lifelong disability with significant comorbidities. The expanded use of exome sequencing has greatly accelerated the genetic characterization of these disorders, and many novel forms continue to be discovered. One such disorder is “neurodevelopmental disorder with hypotonia, neuropathy, and deafness” (MIM# 617519, abbreviated as “NEDHND”) and involves in deleterious variants of *SPTBN4*, named as beta spectrin non-erythrocytic 4. The gene has 12 splice variants and only four of them encode proteins based on Ensembl and Uniport [2, 3]. In general, spectrins are known to play a major role in membrane architecture and stability, and neuronal maintenance, and also serve as binding sites for different receptors such as glutamate EAAT4 transporter [4–6]. The major isoform of SPTBN4 engages with sodium channels and ankyrin-G at nodes of Ranvier and therefore considered to have a likely role in membrane excitability [4–6].

The first study linked *SPTBN4* to NEDHND reported singleton, a hypotonic Kurdish boy from a consanguineous family, with a pathogenic nonsense variant in the gene [7]. A follow-up study examined a cohort of patients (n = 6) and provided extensive molecular view on the gene’s involvement in the disease [8]. Currently, there are only 38 patients (including our 10 cases) reported to carry deleterious variants of *SPTBN4* [4, 7–19] worldwide. These patients have various symptoms of muscle, head and neck, abdominal, skeletal, and nervous systems (OMIM 627519). First reported case presented soon after birth with facial weakness, areflexia, impaired motor development, and feeding problems [7]. Following studies expanded the phenotypic spectrum of the disorder [4, 8–12]. Additional features included complete language impairment, seizure, denervated, atrophic muscles, cortical visual impairment, and deafness. Particularly, two leading studies showed that observed muscle weakness was mainly owing to muscle fiber-type disproportion and motor axonal neuropathy [7, 8].

Due to the clinical heterogeneity, genetic testing is one of the first trier approaches for fast and accurate diagnosis of the syndrome. To our knowledge the literature on NEDHND remains limited, particularly missing the information on the natural history of this ultra rare syndrome. It is essential to gain more knowledge and improve our current understanding in the view of epidemiology and natural history of rare genetic disorders including NEDHND to ameliorate patient care and be able to help better to the suffering patients and their families. Since, well-defined clinical continuum is critical to improve diagnosis and downstream patient care, epidemiological studies and natural history of rare disorders provide a holistic view to explicate clinical and pathobiological aspects of such diseases and related outcomes.

Here, we conducted an extensive search on the previously published cases and sought to elucidate NEDHND cases in our highly consanguineous population. Our approach revealed four deleterious homozygous variants in *SPTBN4* in ten patients from four unrelated consanguineous Saudi families. We presented evidence for one of the variants, a splicing error, at the RNA level and showed segregation of these variants in their respective families. Among the ten patients, phenotypes of three individuals described previously elsewhere [11] and have been extensively studied here. We delineated these Saudi patients’ phenotypes in detail, compared them to previously reported cases, and highlighted atypical features that expand the phenotypic spectrum of the syndrome. Finally, here we aimed to provide the first natural history of NEDHND.

## Materials and methods

### Study materials, sample collections, and ethics

**Fig. 1:**
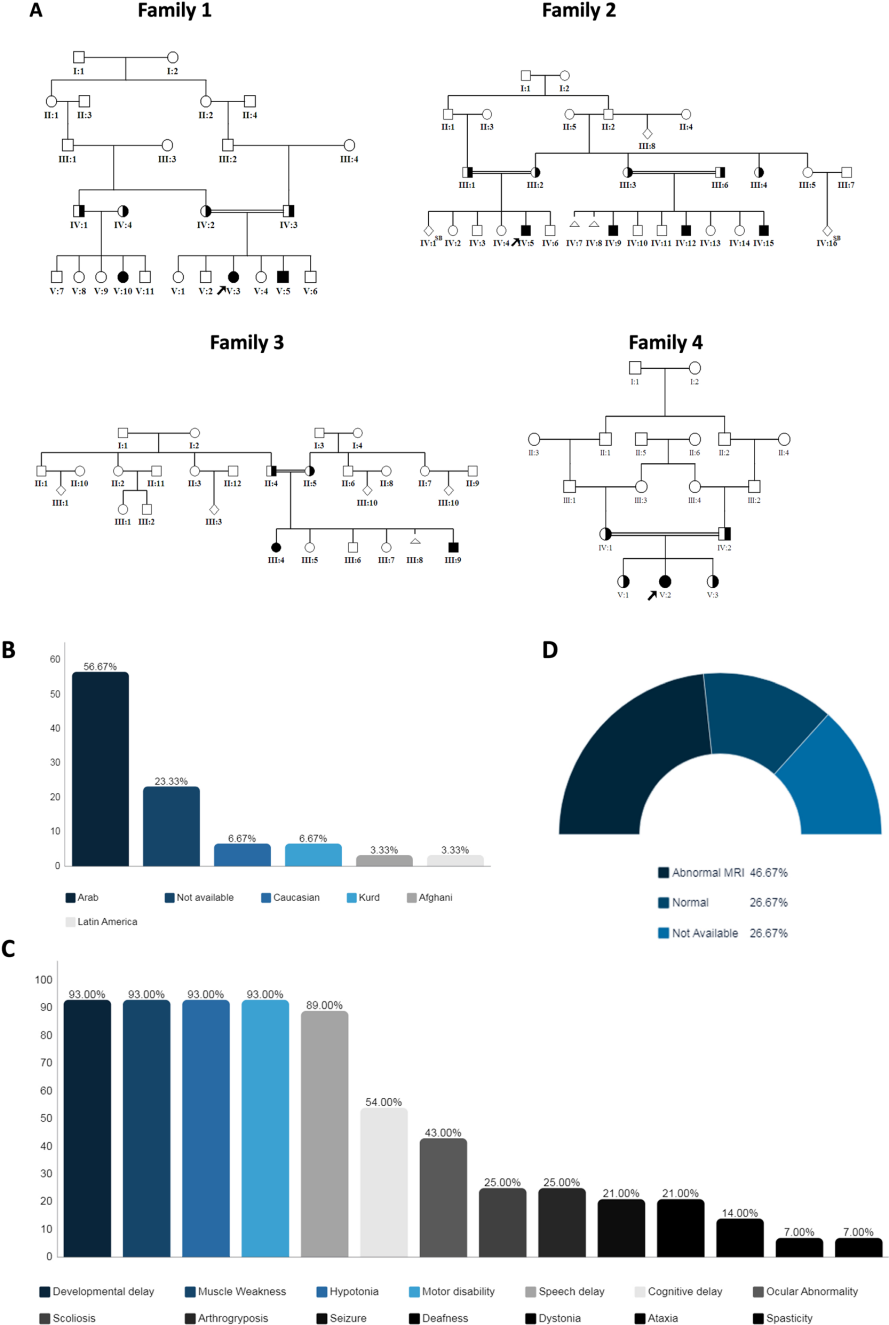

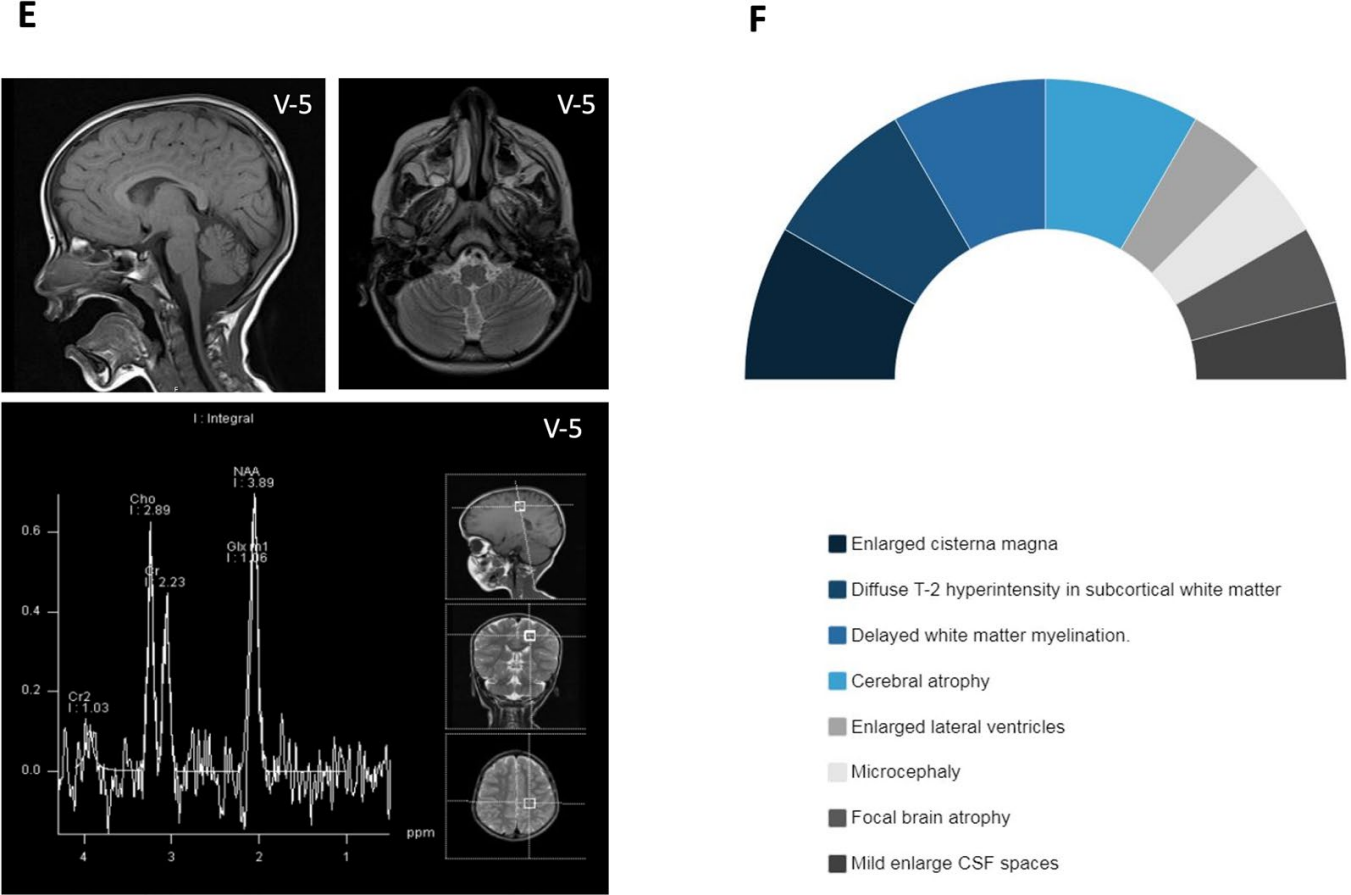
**A** Pedigrees of affected individuals in four consanguineous families. **B** Histograms presenting ethnicities distributions among NEDHND patients. **C** Frequencies of neuromuscular features among NEDHND patients. **D**–**F** Brain MRI abnormalities among NEDHND patients with MRI for patient 2 from our cohort

Ten patients from four consanguineous families were recruited from neurosciences and medical genetics clinics particularly pediatric movement disorders clinic (Fig. 1A). The study was approved by the institutional review boards (IRB) at King Faisal Specialist Hospital and Research Centre (KFSHRC) (RAC# 2120022, 2060035, 2180004) and the participants signed written informed consent forms. Peripheral blood samples (3 ml in EDTA tubes, 3 ml sodium heparin tube) were obtained from the affected and unaffected individuals in the participating families.

### Clinical examination

A comprehensive review was conducted on the medical history and disease presentation of 38 patients, including 31 previously published cases and 7 unreported ones [4, 7–19]. The review includes patients’ ethnicities, parental consanguinity, age of onset, neuromuscular findings, neuroimaging, and genetic results. All relevant information found was reported.

### DNA isolation, and Sanger sequencing

DNA extracted using Gentra® Puregene® DNA Purification Kit (Gentra Systems, Inc. Minneapolis, MN, US). NanoDrop® ND-1000 (NanoDrop Inc., Wilmington, DE, US) was used to measure the nucleic acid quality and quantity. Ensembl and Primer3 web-based tool were used for designing variant specific primer. After optimizing the primer on control DNA, the DNA samples were amplified using standard PCR protocols. Targeted Sanger based sequencing was performed using ABI PRISM 3100 Genetic Analyzer (Applied Biosystems, Foster City, CA, USA).

### Autozygosity analysis

DNA samples were genotyped using Affymetrix axiom chips based on the manufacturer’s assay and protocols. The analysis was done as before [20–23]. Autozygome coordinates were taken into consideration during the WES variant filtering process.

### Gene panel screening

DNA samples were used for a comprehensive gene panel that contains known genes causing recessive ataxias in addition to some other neurological disorders as described before [24]. The panel was run on the Ion Proton next-generation sequencing platform (Thermo Fisher, Waltham, MA, USA).

### Whole exome sequencing (WES) and iterative filtering

WES was undertaken using an Illumina 2500 platform. Libraries were prepared by SureSelect kit that are designed for the Illumina platforms (Agilent Technologies, Santa Clara, CA, US). Captured sequences were read on the Illumina’s platform. Sequence alignment was achieved through in-house pipeline. Generated data was analyzed using publicly and commercially available tools and software. After the analysis of the WES data, the variants were filtered as previously described [20–22, 25].

### RNA isolation and RT-PCR

RNA was extracted from lymphoblastoid cell line (QIAGEN Inc., Valencia, CA, USA). Quality and quantities of the total RNA were determined by measuring the absorbance spectra on a UV/Vis spectrophotometer, the NanoDrop® ND-1000 Spectrophotometer (Nanodrop Inc., Wilmington, DE, USA), and further analyzed by RNA 6000 Nano Assay using 2100 Bioanalyzer (Agilent Technologies, Santa Clara, CA, USA). The un-degraded, high quality RNA was used for RT-PCR experiments. Similarly, Primer3 web tool was utilized for exon spanning primers that ensure successful amplification of exonic regions without inclusion of intronic sequences.

### Neuroimaging and neurophysiological examination

Brain magnetic resonance imaging (MRI) results were found for 22 patients (57.8%). Electroencephalogram (EEG) was performed on 12 patients (31.5%) whereas magnetic resonance spectroscopy (MRS) results were available for 4 patients only (10.5%).

### Muscle biopsy

Muscle biopsy was performed on 8 patients only (21.1%). There wasn't any histopathological work done on the remaining patients.

### Laboratory tests

Renal and hepatic profile, amino acid, CK levels, lactic acid, ammonia, complete blood count (CBC) and other laboratory tests were carried out on some patients.

## Results

### Patients population

Previous studies revealed presence of the disease in Kurdish, Caucasian, Arab, Afghan, Latin American, populations (Fig. 1B, Supplemental Table 1). Interestingly, among the reported cases, there are 17 Arabs including 13 Saudi patients.

### Clinical evaluations

Detailed clinical features of our cohort are presented in Table 1, and supplemental file 1. All the cases reviewed in this study are presented in supplemental Table 1.

**Table 1:** Clinical features of the our cshort in the study

Family		1			2				3		4
Subject		1	2	3	4	5	6	7	8	9	10
Pedigree codes		Family 1, V:3	Family 1, V:5	Family 1, V:10	Family 2, IV:5	Family 2, IV:9	Family 2, IV:12	Family 2, IV:15	Family 3, III:4	Family 3, III:9	Family 4, V:2
Gender		Female	Male	Female	Male	Male	Male	Male	Female	Male	Female
Age at presentation (Year)		4	2	1.10	5	15	−	2	1.4	−	1.8 Y
Consanguinity		+	+	+	+	+	+	+	+	+	+
Ethnicity		Saudi	Saudi	Saudi	Saudi	Saudi	Saudi	Saudi	Saudi	Saudi	Saudi
Mutation	c.DNA change	NM_020971:c.1665+2T>C			NM_020971:c.1217T>C				NM_020971:c.2535-2554del		NM_020971:c.2265G > A
	Amino acid change				p.L406P				p.Gly846Alafs*13		Trp755*
	Mutation type	Splice Site			Missense				Deletion		Nonsense
	Zygozity	Homozygous	Homozygous	Homozygous	Homozygous	Homozygous	Homozygous	Homozygous	Homozygous	Homozygous	Homozygous
Neuromuscular Findings	Developmental delay	+	+	+	+	+	NA	+	+	NA	+
	Muscle weakness	+	+	+	+	+	NA	+	+	NA	+
	Hypotonia	+	+	+	+	+	NA	+	+	NA	+
	Seizure	+	−	+	−	−	NA	−	−	NA	−
	Motor disability	+	+	+	+	+	NA	+	+	NA	+
	Ataxia	+	+	−	−	−	NA	−	−	NA	−
	Scoliosis	+	−	−	−	+	NA	−	−	NA	−
	Cognitive delay	+	+	+	−	+	NA	+	−	NA	+
	Spasticity	−	−	−	+	−	NA	+	−	NA	−
	Speech delay	+	+	+	+	+	NA	+	+	NA	+
	Deafness	+	−	−	−	−	NA	−	−	NA	Mild hearing loss
	Ocular abnormality	−	−	−	−	−	NA	−	Myopia	NA	Myopic astigmatism
	Dystonia	−	−	−	−	−	NA	−	−	NA	−
	Blepharospasm	−	−	−	−	−	NA	−	−	NA	−
	Arthrogryposis	−	−	−	+	−	NA	+	−	NA	−
MRI	Vermian atrophy	+	+	Normal	Normal	Normal	NA	Normal	Normal	NA	Mild
	Enlarged cisterna magna	−	+				NA			NA	Mild
EEG	Epileptiform activity	+	−	−	−	−	NA	−	−	NA	−
	Slow activity	+	+	−	−	−	NA	−	−	NA	−
	Photic stimulation	−	−	−	−	−	NA	−	−	NA	−
MRS	Lactate peak	Normal	N/A	Normal	N/A	N/A	N/A	N/A	N/A	N/A	N/A
	Glutamate peak	Normal		Normal							
	NAA peak	Normal		Normal							
PP	Pneumonia	−	−	−	−	+	N/A	−	−	N/A	+
	Restrictive lung disease	−	−	−	−	+	NA	−	−	NA	+
	Tracheostomy	−	−	−	−	+	NA	−	−	NA	−
Feeding difficulties/gastrostomy tube		−	−	−	−	−	NA	−	−	NA	+
General features	Floppy with head lag	+	−	−	−	−	NA	+	−	NA	+
	Poor dexterity	+	−	−	−	−	NA	−	−	NA	+
	Telecanthus	−	−	−	−	−	NA	−	+	NA	−
	Hypertelorism	−	−	−	−	−	NA	−	+	NA	−
	Flat occiput	−	−	−	−	−	NA	−	+	NA	+
	Prominent low set ears	−	−	−	−	−	NA	−	+	NA	−
	Posteriorly rotated ears	−	−	−	−	−	NA	−	+	NA	−
	Prominent fetal pad	−	−	−	−	−	NA	−	+	NA	−
	High anterior hairline	−	−	−	−	−	NA	−	+	NA	−
	Prominent forehead	−	−	−	−	−	NA	−	+	NA	−
	Sub-gravity neck flexion	−	−	−	+	−	NA	−	−	NA	+
	Tremor	−	−	−	−	−	NA	−	−	NA	−
	Facial weakness	−	−	−	+	−	NA	−	−	NA	+
	Prominint eye	−	−	−	−	−	NA	−	+	NA	−
	Epicanthal fold	−	−	−	+	−	NA	−	−	NA	−
	Lateral gaze limitation	−	−	−	+	−	NA	−	−	NA	−
	Brisk reflex	−	−	−	−	−	NA	+	−	NA	−
	Club paralytic feet	−	−	−	−	+	NA	−	−	NA	−
	Highly arched palate	−	−	−	−	−	NA	−	−	NA	+
	Un-sustained ankle clonus	−	−	−	−	−	NA	+	−	NA	−
MB	Morphology	Normal	N/A	N/A	N/A	N/A	NA	N/A	N/A	NA	NA
	Immunostaining	Normal	N/A	N/A	N/A	N/A	NA	N/A	N/A	NA	NA
Laboratory Tests	Protein and glucose in CSF	Normal	N/A	N/A	N/A	N/A	N/A	N/A	N/A	N/A	NA
	Lactic acid (CSF)	Normal	N/A	N/A	N/A	N/A	N/A	N/A	N/A	N/A	NA
	Lactic acid (Blood)	Normal	N/A	N/A	Normal	Normal	N/A	Normal	Normal	N/A	NA
	Ammonia (Blood)	Normal	N/A	N/A	Normal	Normal	N/A	Normal	Normal	N/A	NA
	Amino acid (CSF)	Glutamic acid, Glutamine, Glycine, Alanine, Valine, Phenylalanine, and Lysine are low. Serine & Tryptophan are high	N/A	N/A	N/A	N/A	N/A	N/A	N/A	N/A	NA
	Amino acid (Blood)	Normal	N/A	N/A	N/A	Normal	N/A	Normal	Normal	N/A	There is increase in ornithine and mild increase in glutamic acid
	Hematology study (CSF)	Normal	N/A	N/A	N/A	N/A	N/A	N/A	N/A	N/A	NA
	CBC	MCV & MCH are Low. Platelets is high	N/A	MCV & MCH are Low. Platelets is high	MCV & MCH are Low. Platelets & RDW high	MCV & MCH are Low. RBC & RDW high	N/A	MCV & MCH are Low	MCV & MCH are Low. Platelets is high	N/A	Normal
	CK	Normal	N/A	N/A	Normal	Normal	N/A	Normal	Normal	N/A	Normal
	Renal profile	Low CO_2_	N/A	Normal	Low CO_2_	Low CO_2_ & Creatinine	N/A	Low CO_2_ & Creatinine	N/A	N/A	CO_2_ and Creatinine are low
	Hepatic profile	At 4 years old ALT was quantified and was decreased, however 9 years later ALT was elevated	N/A	N/A	N/A	N/A	N/A	Normal	N/A	N/A	Normal
	Creatinine, Urine	−	Normal	N/A	N/A	N/A	N/A	N/A	Normal	N/A	NA
	Soluble CD25	−	N/A	N/A	High	N/A	N/A	N/A	N/A	N/A	NA

+Present; − Absent; *EEG* electroencephalogram; *MB* muscle biopsy; *MRI* magnetic resonance imaging; *MRS* magnetic resonance spectroscopy; *NAA* N-acetylaspartate; *N/A* not available; *PP* pulmonary problems

The key features associated with the disease-causing variants in most patients are global developmental delay, neuropathy, and secondary myopathy (Fig. 1C). Nearly all the reported patients experienced muscle weakness, motor disability, hypotonia, and speech delay although recently reported patients also suffer from ADHD, CHD, and autism (no clinical data available) [16, 17, 19]. Ocular abnormalities present in 43% of patients ranging from nystagmus and visual impairment to complete blindness due to optic atrophy. Nevertheless, scoliosis, deafness, and seizure are still present in some of the affected individuals (25%, 21%, and 21%, respectively). Interestingly, ataxia was observed only in a single Saudi family [11]. Facial weakness, lack of head control, and highly arched palate are the most common dysmorphic features observed. Gastrointestinal problems including feeding difficulties, dysphagia, and gastrostomy tube feeding during infancy were reported in 15 patients (54%). Respiratory difficulties were seen in 17 patients (61%). Ten of the 17 patients suffered from recurrent pneumonia, which developed only in 2 of them to restrictive lung disease and the patient needed tracheostomy and home ventilator. Two of the 17 patients died at age of 14 months and 3 years due to respiratory failure. Cardiovascular abnormalities were seen in two patients only.

### Neuroimaging and neurophysiological examination

EEG data was available for 12 patients; results of 5 patients showed abnormal EEG the remaining 7 patients were normal. MRI results were available for 22 patients, 14 of them had abnormal MRI findings (Fig. 1D). Such abnormalities included vermian atrophy, diffuse T2-hyperintensity, mildly enlarged CSF spaces, cerebral atrophy, and white matter abnormalities. The remaining 8 patients revealed normal MRI findings. MRS data were available for 4 patients; results showed abnormal metabolites levels with nonspecific lipid and lactate and high glutamate peak in one patient and normal findings in the other three patients (Fig. 1E and F).

### Muscle biopsy

Muscle biopsy morphology results were available for 8 reported cases. The pathology data revealed presence of muscle fiber atrophy (diameter reduction in fiber atrophy type 1) that was encountered more than fiber atrophy type 2 among 5 individuals. In contrast, muscle biopsy from a reported patient at age of 2 years, showed mild muscle fiber hypertrophy [13]. Neurogenic changes were observed in 2 cases. However, one patient from our cohort showed normal findings.

### Genetic cause identification and molecular analysis

**Fig. 2:**
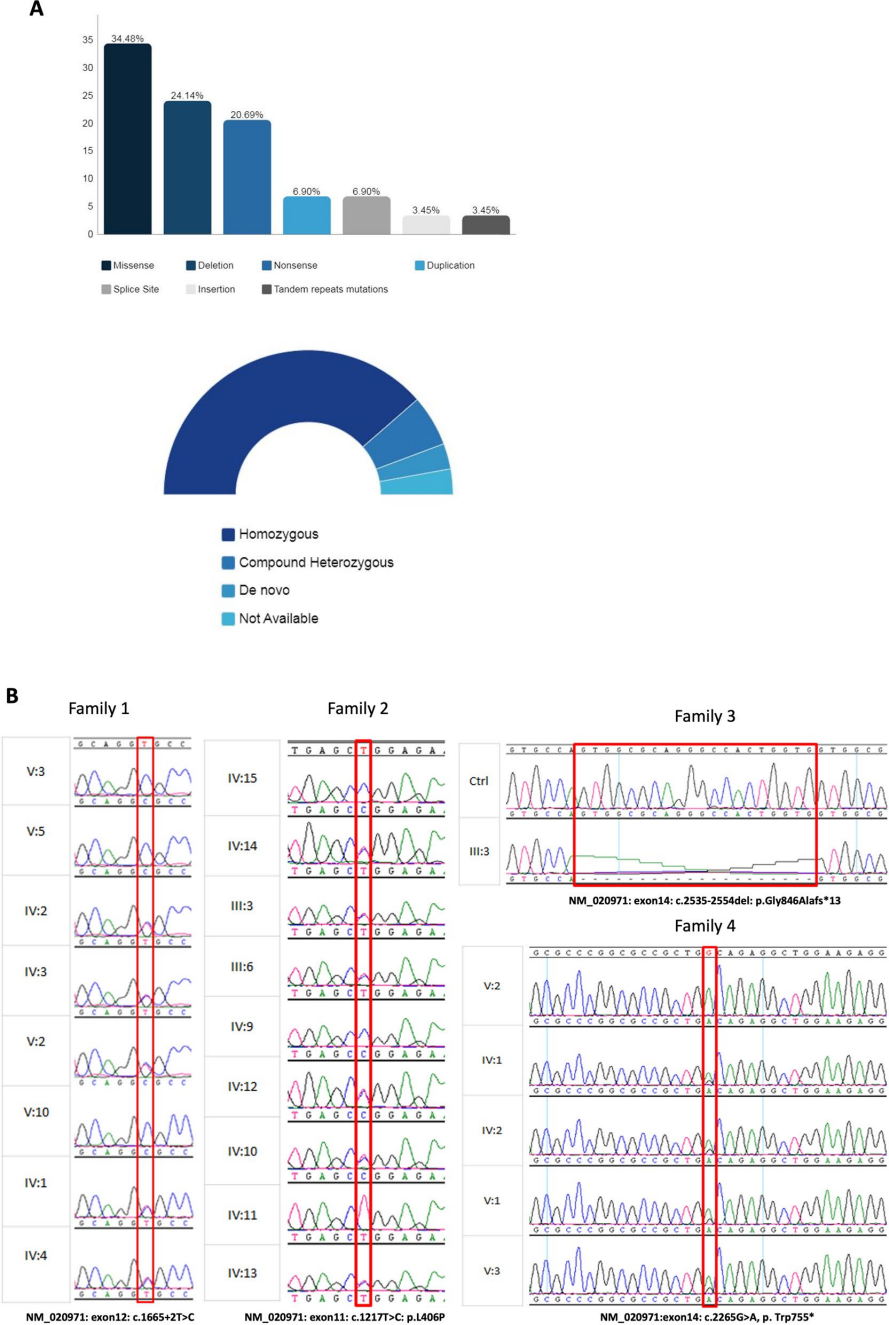

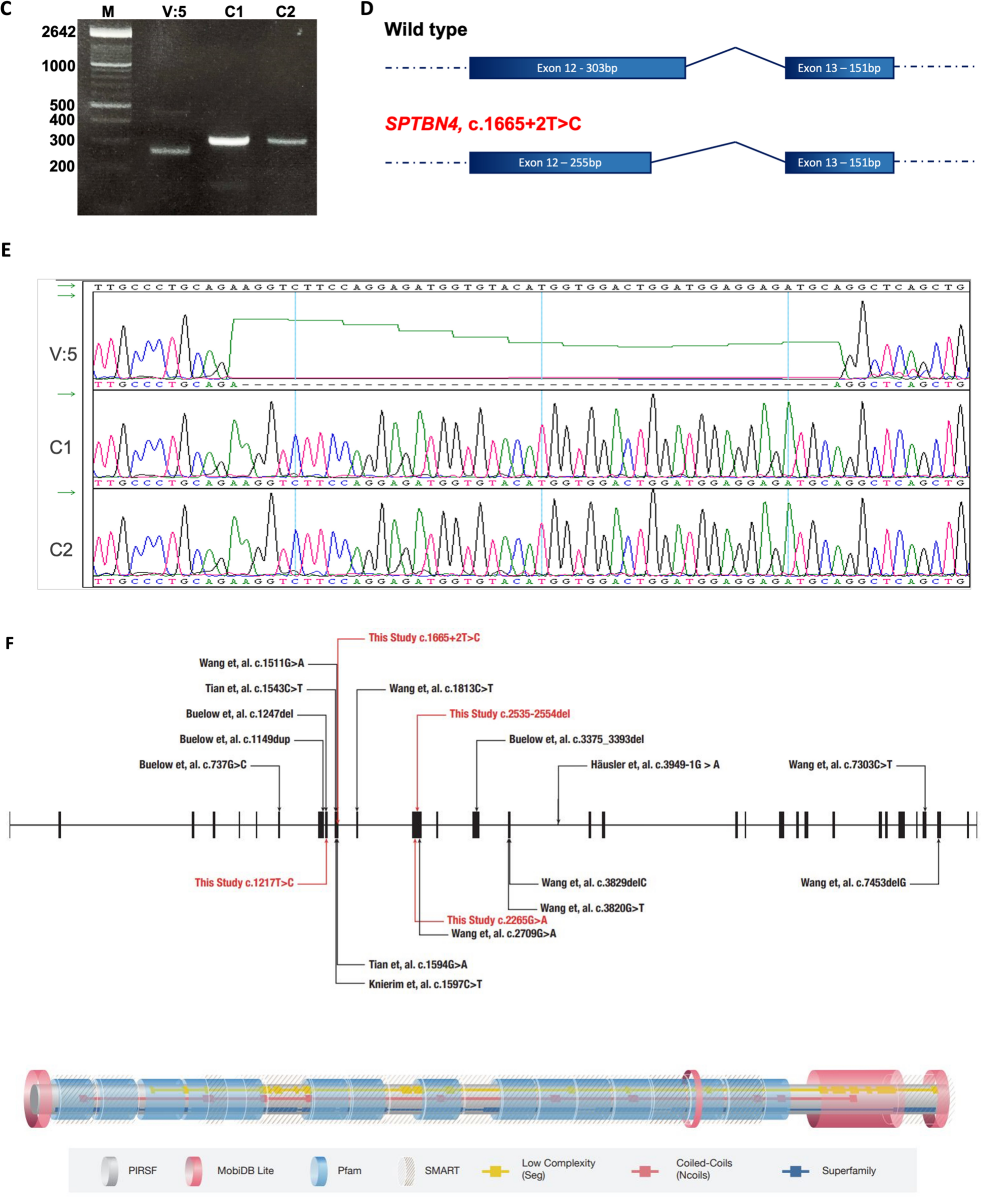
**A** Histograms presenting types of genetic variations reported in all NEDHND patients. **B** Sanger sequencing electropherogram showing *SPTBN4* variants in four families (c.1665 +2T>C, c.1217T>C, c.2535-2554del and c.2265G> A). **C** RT-PCR was performed on RNA extracted from lymphoblastoid cell line samples, result was visualized using 2% agarose gel electrophoresis. **D** Representation of wild type and abnormal cDNA. **E** Sanger Sequencing results showed splice mutation with an abnormal product (48 bp). **F** SPTBN4 Model illustration. The model shows reported variants, exons (Upper panel) and protein domains (Lower panel) on the model according to Ensembl

Thirty-one different *SPTBN4* pathological variants have been discovered in 38 patients. Deleterious variants include missense, nonsense, splice site, deletion, insertion, duplication, and tandem repeats variants with homozygous or compound heterozygous status (Fig. 2A). Homozygous changes dominate among the variations, whereas compound heterozygous occurred in 4 patients only. Two de novo mutations have been reported in two patients, one of them is autistic patient with a variant in the tandem repeats (AGCGGGCGC) [15, 16]. For our cohort, we genetically and clinically screened more than 200 families having affected individuals with neurodevelopmental manifestations and neurological abnormalities. The screening (WES and gene panel) revealed 4 families with 10 patients with *SPTBN4* deleterious variants. Each family carried a separate variant (Table 1). Among the variants, three (NM_020971: c.1217T>C, c.1665 +2T>C, and c.2535-2554del) were previously reported in another study [11]. However, c.2265G> A, (NM_020971: exon14: p.Trp755*) is a novel variant. Since the two patients (V:3 and V:5) in family 1 have ataxia, V:3 was tested on an ataxia‐related gene panel (inclusive of 113 ataxia genes) at Mayo clinics. The same sample was also run on an in-house gene panel [24]. These efforts did not reveal any positive hit on any known ataxia gene since the panels did not include *SPTBN4*. The confirmatory Sanger sequencing pointed a complete segregation of these four variants in each family (Fig. 2B).

To understand the effect of the splicing variant (detected in family 1), we performed RT-PCR on the extracted RNA sample (V:5, family 1). The results revealed that the variant leads to an aberrant mRNA transcript with a shorter product visualized on 2% agarose gel (Fig. 2C). Sanger sequencing of the RT-PCR product displayed a smaller exon (exon 12) that had a 48 bp skipped region due to likely alternative 5’ splice site (Fig. 2C, D and E).

### Laboratory tests

Protein, glucose, lactic acid, and hematological analytes in the cerebral spinal fluid (CSF) of patient 1 (V:3) were all within normal ranges. However, the CSF amino acid profile revealed low levels of glutamic acid, glutamine, glycine, alanine, valine, phenylalanine, and lysine and high levels of serine and tryptophan. Blood lactic acid, ammonia, and Creatine kinase (CK) were normal for all tested patients (8 out of 8 patients for lactic acid and ammonia, 10 out of 10 patients for CK). Five patients' renal profiles showed low CO2 levels (patients 1, 4, 5, 7 and 10). In addition to lower CO_2_, patients 5, 7 and 10 showed reduced creatinine levels. Alanine transaminase (ALT) was quantified for patients 1, 7 and 10. For patient 1, ALT measurement (at 4 years old) result showed decreased ALT level; however, 9 years later ALT was elevated. The other two patients’ hepatic profile was normal. Urine creatinine was measured for patients 2 and 8 and had a normal level. Soluble CD25 was measured for patient 4 only and found to be at high level.

## Discussion

NEDHND is an autosomal recessive disorder first described by Knierim et al. in 2017 [7]. It is caused by genetic variations in spectrin beta chain, non-erythrocytic 4 gene (*SPTBN4*). Here, we provide the first report describing the natural history of patients with “neurodevelopmental disorder with hypotonia, neuropathy, and deafness” (NEDHND, OMIM: 617519), and present clinical descriptions of ten Saudi patients, the largest cohort from a consanguineous population for the gene. Additionally, we report a novel truncation variant and evidence for a novel splice variant. We show that this particular variant causes an aberrant, shorter, transcript in the patient’s blood.

NEDHND has a high incidence rate in consanguineous populations among different ethnicities, where more than 66% of reported cases have known consanguinity status. First human mutation, a nonsense variant, was identified in a Kurdish patient belonging to a first-degree consanguineous Kurdish family through combination of autozygosity mapping and whole exome sequencing [7].Interestingly, clinical symptoms (such as myopathy, deafness, and neuropathy) observed in the affected boy resembled quivering mouse model that were spontaneously arising series of mutant mice during late 1950s presented with deafness, progressive ataxia with hindlimb paralysis, and tremor symptoms [26]. Based on the resemblance, the authors investigated the muscle of the animals as well as muscles from the patients both of which were absent for SPTBN4 expression [7].

Arabs harbor significantly higher number of patients (n = 17) and 13 of them being Saudis. The remaining patients in the literature with other ethnicities reach only ten patients. However, there are some patients (n = 11) whose ethnicity was not mentioned in the literature. No sex preference was observed; where 36.84% of reported patients are males and 44.73% are females. Gender was not specified for 7 patients (18.42%).

Neurologic features dominate NEDHND symptoms. Such features are indiscriminately observed among different ethnicities including Saudis although life expectancy and disease progression show considerable variation among the patients. Symptoms manifest mostly during infancy to early childhood with static or slow progression. Among all cases (n = 38), only 29 patients’ data was provided and the age at presentation was mostly at birth (n = 15) (Supplemental Table 1). In our cohort, the age at presentation to medical services was available for 8 patients and varied between 13 months and 15 years (Table 1). Interestingly, among all the patients including ours, signs of developmental delay or motor dysfunction were noticed by parents during early childhood; only two cases have late disease onset at the ages of 48 and 60 years old [10].

The phenotypic spectrum of *SPTBN4* mutations ranges from motor dysfunction to severe intellectual disability [4, 7–19]. Ataxia was never reported in any NEDHND patients; however, two patients in our cohort suffered from ataxia (patients V:3 and V:5 in family 1). It is worth mentioning that their maternal cousin (patient V:10) didn’t show ataxia symptoms, it could be due to her young age (last follow up was at age of 3 years old). Although no NEDHND patients have ever reported having ataxia, *SPTBN4* mutant mice demonstrated ataxia [26–28]. Moreover, structural and functional homologies between SPTBN4 in mice and human were reported [29].

The most recent reported patients with *SPTBN4 *variants suffer from attention-deficit hyperactivity disorder (ADHD), autism, and congenital heart defects (CHDs) with no other clinical data available [16–19]. Variants in *SPTBN4* noncoding regions and tandem repeats (TRs) were detected in ADHD and autism patients. Cardiac symptoms were reported in two patients [13, 19], the first one experienced common neuromuscular features in NEDHND patients in addition to hypertophic cardiomyopathy of non-obstructive type. Furthermore, he demonstrated inactivity osteoporosis and bone fracture which was reported for the first time. The second patient was reported to have congenital heart defects with no data available about neuromuscular symptoms. βIV-spectrin is found in cardiomyocytes and it is an essential element to maintain the function of voltage-gated sodium channel. Abnormal expression of *SPTBN4* mRNA disturbs Na^+^ current contributing along with other factors to cardiomyopathy. Further, mutant βIV-spectrin mice reported reduced cardiac function as a result of improper cell growth [30–32].

It is important to note that βIV-spectrin protein comprises important domains such as calponin homology (CH) and spectrin repeats domains (Fig. 2F). Looking at the types and specific location of the reported mutations, more than 70% of *SPTBN4* variants are located in spectrin repeat domains. Missense variants were detected in 11 patients (4 of them are from our cohort). Clinical data for 3 of them is not available. Most of these variants (*n* = 6) cluster in spectrin repeat domains. However, for the remaining patients (n = 8), only 5 patients encounter much milder symptoms or late disease onset. The patients in our cohort are able to walk and sit independently; although, they use wheelchair in their daily life. They talk in full sentences and attend special education schools. It is worth to mention that remaining 3 patients with missense variants had significantly more severe symptoms. The missense variants of these patients are found on CH and VR domains. Among them, one compound heterozygous variant (paternal or maternal allele) is located in PH domain. The variability in the disease presentation suggests that other factors may play a role in pathogenesis of NEDHND along with βIV-spectrin such as ankyrin G protein [33].

Among all the reported variants there were only c.1665+2T>C and c.3949-1G>A (NM_020971) that cause aberrant splicing and, interestingly, both were reported among Arabs. In 2020, Häusler et al. (2020) showed a recessive splicing variant in two siblings, a female, and a male, from first-degree consanguineous carrier parents [4]. Disease onset starts at birth with delayed motor development with no regression of motor capabilities. The patients characterized by muscular hypotonia and axonal neuropathy. The variant led to in-frame skipping of exon 19. The index case and her brother showed hypotonia, mild dysarthria, and predominantly motor neuropathy that are similar to the phenotypes in our patients. In contrast to our findings, the index case showed a normal cognitive function. She attended regular school, was able to walk at age of 5 with the aid of walker for short distances unlike our case who is wheelchair bound [4]. Both cases showed speech impairment and abnormal MRI pictures. Bilateral ptosis was reported in the index case [4] while our patient showed normal ocular function. In Häusler et al. (2020) study, muscle biopsy showed neurogenic changes; on the other hand, biopsy was normal in our patient. The absence of seizures, hearing defects, scoliosis, and intellectual delay are the main clinical differences between the cases reported in Häusler et.al. (2020) and our cohort. In our cases (patient 1, 2 and 3), splice site variant results in 48 bp deletions in exon 12 unlike the splicing variant reported in Häusler et al. (2020) study which showed a complete absence of exon 19. This difference could be attributed to the presence of residual protein activity rather than complete loss of function as suggested by Häusler et al. (2020) [4].

It is noteworthy to mention a recent study by Sert et al. (2024) [34]. This study highlights β1 spectrin's role in maintaining neuromuscular junction Na⁺ channels, ensuring robust neuromuscular transmission as previously shown by the same group (Zhang et al. 2021) [35]. Such role might partially compensate for β4 spectrin loss in neurons affecting neuromuscular signaling, though the effects are not complete. The study also demonstrates that β4 spectrin is not expressed in human and mice skeletal muscle or NMJs (tested at mRNA/protein levels as well as using knockout mice), hence suggesting that β4 spectrin's critical roles are neurogenic, particularly in maintaining ion channel clustering at neuronal axon initial segments and nodes of Ranvier. Relatedly, myopathy observed in individuals with NEDHND likely arises from disrupted neuromuscular transmission due to neuronal dysfunction, rather than direct muscle pathology, hence, implicating predominantly affecting neuronal pathways emphasizing the need for neurophysiological assessments and management strategies in affected individuals. In other words, contrary to prior assumptions, β4 spectrin is likely not required for skeletal muscle health or function; myopathy previously associated with *SPTBN4* mutations is possibly neurogenic rather than myogenic.

## Conclusions

In summary, our study presents natural history of NEDHND syndrome, reviews previously reported Saudi patients with the disease, reports additional 7 new patients from 4 Saudi families, and shows experimental evidence of an aberrant splicing variant of *SPTBN4* and its classification on the diagnostic setting based on ACMG guidelines. Additionally, our study explores clinical features of the new patients and expands the phenotypic spectrum of the disease. To our knowledge, this is the first systematic analysis focusing on NEDHND in the Gulf and Middle East regions and reviews the largest cohort with the disease among Arabs. Since, the genetic counseling and related preimplantation genetic diagnosis and in vitro fertilization studies are highly popular in Saudi Arabia due to the frequent appearance of genetic rare disorders in its consanguineous population, these data are critical for genetic counselling of the inflicted families as well as carrier testing programs in our country and may also help future directions for disease treatment approaches and trials.

## Supplementary Information

Additional file 1.

Additional file 2.

## Data Availability

The authors confirm that the data supporting the findings of this study are available within the article [and/or] its supplementary materials.

## Abbreviations

ABR: Auditory brainstem response
ACMG: Advisory committee on genetic modification
ADHD: Attention deficit hyperactivity disorder
AE: Age-equivalent
ALT: Alanin transferase
CD25: IL-2 receptor
CBC: Complete blood count
CH: Calponin homology
CHD: Congenital heart defects
CK: Creatine kinase
CO2: Carbondioxide
CSF: Cerebrospinal fluid
DBS: Dry blood spot
DTR: Deep tendon reflexes
EDTA: Ethylenediaminetetraacetic acid
EEG: Electroencephalogram
IRB: Institutional review board
KFSHRC: King Faisal Specialist Hospital and Research Centre
MRI: Magnetic resonance imaging
MRS: Magnetic resonance spectroscopy
NBS: Newborn screening
NEDHND: Neurodevelopmental disorder with hypotonia, neuropathy, and deafness
PH: Pleckstrin homology
RAC: Research advisory council
RT-PCR: Reverse transcription polymerase chain reaction
SR: Spectrin repeats
TR: Tandem repeats
VEP: Visual evoked potentials
VR: Variable repeats
WES: Whole exome sequencing
