# Supplementary material for: Natural history of SPTBN4-related neurodevelopmental disorder with hypotonia, neuropathy, and deafness
Source: Orphanet J Rare Dis. 2025 Aug 8;20:415. doi: 10.1186/s13023-025-03810-4 (PMC12335179; doi:10.1186/s13023-025-03810-4)
Supplement: Supplementary file 1 — Additional file 1. [file 13023_2025_3810_MOESM1_ESM.docx]

**Supplemental file 1. Detailed clinical descriptions**

**Patients 1 and 2 (Family 1, V:3 and V: 5)** are sister and brother with known history of cerebellar ataxia. They have four healthy siblings in the main branch. Patient 3 is a cousin from another branch of the family. Both siblings, patients 1 and 2, experience similar clinical manifestations. They were born with a significant motor disability secondary to hypotonia, ataxia, and language impairment. Around the age of 5 years, the diagnosis of cerebellar ataxia with global developmental delay became definitive in both patients. There was no history of visual abnormalities observed in them. The girl, the index case, was floppy with head lag since her infancy. Progressively, she has had poor dexterity and severe dysarthria. She is wheelchair-bound and was never able to walk. She suffered from seizures that were controlled by medication. Her ocular examination was normal with no nystagmus. At age of I year old a muscle biopsy was obtained and subjected to immunohistochemistry panel of muscle-associated proteins including spectrin, beta-dystroglycan, dystrophins, merosin, emerin, myosins, desmin, and alpha, beta, gamma, and delta sarcoglycans. Results showed normal proteins expression. At the age of 9, she was diagnosed with myopathy and significant scoliosis. She suffers from deafness. Leiter International Performance Scale IQ test revealed normal cognitive abilities. Her younger brother was first examined at the age of 1 year. He appeared to be similarly affected at that time. The boy is moderately handicapped with motor disability as well as language difficulty. Unlike his sister, he did not experience any seizures. The patients were enrolled in a special school and a comprehensive rehabilitation program. They have shown improvement in their receptive and expressive language skills. However, they still have difficulties in daily life activities and are completely dependent on their family. Both patients were subjected to a standardized neurologic examination. Brain MRI showed mild midline vermian atrophy (Figure 1C) while EEG revealed intermittent slow activity suggesting mild cortical dysfunction and nonspecific encephalopathy for both patients. The latest clinical assessment showed that the patients had slow progression of their symptoms.

**Patient 3 (Family 1, V: 10)** is maternal cousin of the first two patients. She suffers from similar clinical presentation as her cousins. She was born with hypotonia and muscle weakness resulted in delayed development. She is unable to stand or walk dependently. She showed speech difficulties; however, vision and hearing functions are normal.

**Patient 4 (Family 2, IV: 5)** is the index case in a Saudi consanguineous family with a significant history of neuromuscular diseases with several affected members. He was born with muscle weakness and hypotonia that led to progressive developmental delay. Additionally, he has fixed contractures of the achilles, hamstring and adductor muscles which resulted in bilateral hip dislocation making him wheelchair-bound. He has speech difficulties with normal cognitive functions. His physical examination showed appendicular spasticity with predominance in the lower extremities, sub-gravity neck flexion, and brisk reflex. He has mild facial weakness with epicanthal fold. He suffers from mild limitation in the lateral gaze of the eyes with no ptosis. He has no seizures, nor ataxia. His hearing and vision were normal. Muscle biopsy was obtained and showed mild myopathic changes with moderate increase in variation of fiber size and some internal nuclei however, no obvious dystrophic changes observed. On the other hand, immunostaining revealed absence of dystrophin-2 (C terminus) antibodies staining. Brain MRI revealed normal findings.

**Patients 5, 6, and 7 (Family 2, IV: 9, IV:12, and IV: 15, respectively)** are brothers with other healthy siblings. The patient 5 (Family 2, IV: 9) has developmental delay, kyphoscoliotic deformity, and bilateral club paralytic feet deformities. He is wheelchair-bound and was never able to walk. He was subjected to thoracolumbar Spine X-ray and MRI. Images showed no tethering of the cord, however significant long-curve right convexity scoliosis of the thoracolumbar spine was observed with no associated cord anomaly. Pulmonary evaluation suggested he has moderate risk of restrictive lung disease. At 16 years old, he was hospitalized due to pneumonia. Subsequently, he underwent tracheostomy and needed repeated suction. Currently, he is tracheostomy dependent with progressive restrictive lung disease. Patient 6 (Family 2, IV: 12) was not clinically examined at our hospital and there is no clinical details about him. The family agreed to collect blood from him for genetic testing only. Patient 7 (Family 2, IV: 15) is the index case in this family and the youngest affected sibling. He has developmental delay secondary to axial hypotonia and axial motor weakness since his infancy. He is wheelchair-bound and suffers from mild head lag. He has no dysmorphic skeletal features indicating specific dysplasia. He has mild cognitive delay and language difficulty; however, his vision and hearing are normal. His physical examination showed appendicular spasticity with predominance in the lower extremities and Mild prominent eyes bilaterally with no ptosis. He never experienced seizures. Standard Muscular examination showed bilateral muscle atrophy in hands and legs with spastic limbs and mild ankle contracture. Moreover, deep tendon reflexes were brisk; with bilateral un-sustained ankle clonus was observed. Electromyography (EMG) from tibialis anterior and from vastus medialis on the right side showed chronic neurogenic changes. The patient also has brisk DTRs and ankle clonus. The picture suggests chronic motor axonal neuropathy with pyramidal changes as well as cognitive delay. Brain MRI was unremarkable.

**Patient 8 and 9 (Family 3, III: 4 and III: 9)** are sister and brother from consanguineous family experienced central and peripheral hypotonia, and psychomotor delay. She suffers from dysmorphic features includes: high anterior hairline, prominent forehead, hypertelorism, telecanthus, long eyelashes, flat occiput, posteriorly rotated low set ears, which are relatively prominent, mild clinodactyly, deep seated nails and 1 small tiny area of hypopigmentation on the back in addition to prominent fetal pads. Optical examination showed moderate myopic changes. Brain MRI was normal.

**Patient 9 (Family 3, III: 9)** is handicapped with similar manifestation as his sister and had severe global developmental delay with head lag according to his parents. Similar to patient 6, he was not clinically examined at our hospital and there is no clinical information about him. The family only approved to collect blood samples from him to get genetic testing done.

**Patient 10 (Family 4, V:2)** is a full-term girl, her parents are first degree cousins with negative family history for similar symptoms . She suffered from a global developmental delay with hypotonia. She was clinically evaluated at 22 months old. A head lag was demonstrated secondary to a profound motor delay. She had a minimum upper limb movement where she could reach objects with no lower limb movement. She presented with microcephaly, mild facial weakness and high arched palate. She was visually interactive with myopic astigmatism. She had speech delay with mild hearing loss. The parents reported a history of irritability and lethargy. She suffered from a persistent rhinorrhea, cough, sputum production, increased work of breathing and multiple respiratory infections. She experienced progressive oropharyngeal dysphagia and aspiration illustrated by poor oral control and recurrent episodes of chocking during feeding for solid and soft food as well as for liquids. Therefor a [laparoscopic nissen fundoplication and gastrostomy tube was inserted. EEG indicates moderate to severe cerebral dysfunction with no seizure activity. The nerve conduction study of the lower extremities is normal with no evidence of neuromuscular junction disorder based on the repetitive nerve stimulation. However, needle EMG shows some finding that is more suggestive of myopathy based on the morphology of the motor unit and the recruitment pattern. Patient suffered from hiatal hernia and underwent surgical repair. Patient died at age of 3 years old as a result of hypoxic respiratory failure secondary to aspiration pneumonia due to oropharyngeal dysfunction.](https://www.youtube.com/watch?v=zWorYnHhjiM)
